# Metabolic and transcriptional analysis of tuber expansion in *Curcuma kwangsiensis*

**DOI:** 10.1038/s41598-024-84763-9

**Published:** 2025-01-10

**Authors:** Yunyi Zhou, Lixiang Yao, Yueying Xie, Baoyou Huang, Ying Li, Xueyan Huang, Liying Yu, Chunliu Pan

**Affiliations:** 1 Guangxi TCM Resources General Survey and Data Collection Key Laboratory/ the Center for Phylogeny and Evolution of Medicinal Plants, Guangxi Botanical Garden of Medicinal Plants, Nanning, China; 2 National Center for TCM Inheritance and Innovation, Guangxi Botanical Garden of Medicinal Plants, Nanning, China

**Keywords:** Metabolites, Transcripts, Molecular mechanism, *Curcuma Kwangsiensis*, Tuber expansion development, Plant morphogenesis, Plant molecular biology

## Abstract

The tubers of *Curcuma kwangsiensis* are regarded as an important medicinal material in China. In *C. kwangsiensis* cultivation, tuber expansion is key to yield and quality, but the regulatory mechanisms are not well understood. In this study, metabolomic and transcriptomic analyses were conducted to elucidate the mechanism underlying tuber expansion development. The results showed that auxin (IAA), jasmonic acid (JA), gibberellin (GA_3)_, ethylene (ETH), and brassinolide (BR) levels increased during tuber expansion development. Metabolomic analysis showed that 197 differentially accumulated metabolites (DAMs) accumulated during tuber expansion development and these also play important roles in the accumulation of carbohydrates and secondary metabolites. 6962 differentially expressed genes (DEGs) were enriched in plant hormone signal transduction, starch and sucrose metabolism, linoleic acid metabolism, MAPK signaling pathway as well as sesquiterpenoid and triterpenoid biosynthesis. Comprehensive analysis revealed that DEGs and DAMs of plant hormone signal transduction, ABC transporters and biosynthesis of phenylpropanoids and terpenoids are critical pathways in regulating tuber expansion. In addition, some transcription factors (*ARF*, *C2H2*, *C3H*, *NAC*, *bHLH*, *GRAS* and *WRKY*) as well as hub genes (*HDS*, *HMGR*, *ARF7*, *PP2CA*, *PAL* and *CCOMT*) are also involved in this process. This study lays a theoretical basis for the molecular mechanism of tuber expansion in *C. kwangsiensis*.

**Supplementary Information:**

The online version contains supplementary material available at 10.1038/s41598-024-84763-9.

## Introduction

*Curcuma kwangsiensis* S. G. Lee et C. F. Liang, belonging to genus *Curcuma*, Zingiberaceae family, is a traditional Chinese medicinal herb widely cultivated in southwestern China. The rhizomes and tubers of *C. kwangsiensis* are recognized as one of the sources of Ezhu and Yujin, respectively, as documented in the Chinese Pharmacopoeia (2020 edition)^1^, with distinct therapeutic properties. Compared to rhizomes, tubers exhibit a higher yield, thus making tuber size a critical factor in deterimining the planting efficiency of *C*. *kwangsiensis*. The active ingredients of *C*. *kwangsiensis* tubers are volatile oils composed of structurally diverse sesquiterpenoid compounds, such as germacrone, curzerenone, beta-elemen and curcumenol^2,3^, offering anticancer, anti-inflammatory, antioxidant, and hepatoprotective benefits^4,5^. During artificial cultivation, the cultivated tubers display significant size variation, leading to low yield and disparity qualities^6^. Therefore, the mechanisms underlying the development of *C. kwangsiensis* tubers and the biosynthesis pathways of their metabolites is crucial for enhancing both their quality and yield, and also will provide a fundamental basis for optimizing production strategies.

Tuber plants are characterized by their expansion of underground storage organs. Tuber development mainly includes initiation, expansion, and maturation stages, accompanied by the accumulation of metabolites (starch, storage proteins and other metabolites)^7–9^. Great efforts have been made to explore tuber development commonly caused by environmental and endogenous signals^10^.Potato and yam are among the most important tuber crops, and their tubers are expansion stems originating from belowground stolon and hypocotyls, respectively. The short-day treatment accelerated tuber formation in potato and promoted tuber initiation and expansion growth in yam, but the responses varied among species and cultivars^11–13^. Endogenous phytohormones are involved in tuber developmental processes. High gibberellins (GA) and abscisic acid (ABA) levels inhibit tuber initiation and retard expansion in potato^9^, and GA, ABA, trans-zeatin (ZR) and jasmonic acid (JA) are also involved in accelerating tuber expansion growth of yam^7^. The *StGA2ox1* (GA2-oxidase) expression level was upregulated in the subapical zone of the stolon and growing tuber before tuber expansion, which the ABRE-binding factor genes *StABF4* and *StABF2* positively regulate tuber induction, suggesting these genes play a role in tuber development^14,15^. *DELLA* (DELLA protein), *Aux/IAA* (auxin influx carrier/auxin-responsive protein), *ARF* (auxin response factors) and *SAUR* (small auxin up RNA) genes were significantly abundant in the tuber expansion stage^16^. In medicinal plants, enlarged tubers act as crucial medicinal organs accumulating abundant bioactive ingredients. There are several genes involved in *C. longa* tuber development were significantly enriched in the carbohydrate metabolism, phytohormone signaling, phenylpropanoid and transporter pathway, include *IAA6* (auxin-responsive protein 6), *PYR1* (abscisic acid receptor 1), *ARF17*, *ABI5* (abscisic acid insensitive 5), *GID1*, *MYC2/4*, *SWEET* (sugars will eventually be exported transporters), *STP5* (sugar transport protein 5), *PAL*, *C4H* (cinnamate 4-hydroxylase), *4CL* (4-coumarate-CoA ligase), *CCOMT* (coffee acyl coenzyme A-3-O-methyl transferase) genes^17^. The terpenoid biosynthesis, plant hormone signal transduction, phenylpropanoid biosynthesis, flavonoid biosynthesis, and other synthesis pathways are found in tubers of *C. wenyujin*^18^. Nonetheless, there are still significant gaps in our knowledge of tuber development, particularly its molecular mechanisms in medicinal plants.

The analysis of the tuber development process of *C. kwangsiensis* is beneficial for improving their yield and quality during cultivation^6^. *C. kwangsiensis* tubers have active sesquiterpenoid compounds. Numerous studies have shown that sesquiterpenoids comprise an important component of their pharmacological activity^4,19^, and sesquiterpenoid synthesis has been identified by studying terpenoid biosynthesis^20,21^. The mevalonic acid (MVA) and 2-C-methyl-D-erythritol 4-phosphate (MEP) pathways are two synthetic routes to the biosynthesis of terpenoids. 3-hydroxy-3-methylglutaryl coenzyme A reductase (HMGR), 1-deoxy-D-xylulose-5-phosphate synthase (DXS), 1-deoxy-D-xylulose-5-phosphate reductoisomerase (DXR), isoprene pyrophosphate synthase and terpene synthase (TPS), as well as other enzymes, have been reported to be key enzymes in terpenoid biosynthesis^20,22^.In addition, the genes encoding transcription factor (TFs), including *bHLH* (basic helix-loop-helix), *WRKY*, *NAC*, *MYB* (myeloblastosis), *GRAS* and *ARF*, and ATP-binding cassette transporter (ABC transporter), regulated terpenoid biosynthesis during tissue development in *C. wenyujin*, *Aconitum heterophyllum*, *Agriophyllum squarrosum* and celery^18,23–25^. However, the specific roles of the terpenoid-related gene-regulating network are still lacking and impede our understanding of the tuber development process in *C. kwangsiensis*.

Recently, the genes and metabolites involved in the development of enlarged tissues in medicinal plants have been investigated through an intergrative approach combining metabolomics and transcriptomics, such as *Dioscorea polystachya* tubers^26^, *Tetrastigma hemsleyanum* tuberous root^27^, *Panax notoginseng* taproots^28^. *C. kwangsiensis* tuber possesses high medicinal value and economic benefits, with size influencing both the yield and quality^6^. The analysis of the tuber development process of *C. kwangsiensis* is beneficial for improving their yield and quality during cultivation. Understanding the metabolites and constructing the molecular mechanism of tuber expansion development can provide an important basis for its high-yield breeding and the synthesis of quality components in *C. kwangsiensis*. In this study, the initiation and expansion stages of tuber development were selected for metabolomic and transcriptomic analysis. This study aimed to uncover the key molecular network during tuber development in *C. kwangsiensis*.

## Results

### Morphological investigation of tuber expansion

The tuber characteristics of *C. kwangsiensis* were significantly different at the initiation and expansion stages (ET and MT) (Fig. S1C). Hormonal analysis revealed distinct profiles at these two stages. ZR, ABA and salicylic acid (SA) were high at the initiation stage, while IAA, JA, GA, ethylene (ETH), SA and brassinolide (BR) were significantly accumulated at the expansion stage (Fig. S1D). The expansion of the tubers might be related to the hormonal level variations.

## Metabolites identification and analysis

**Fig. 1:**
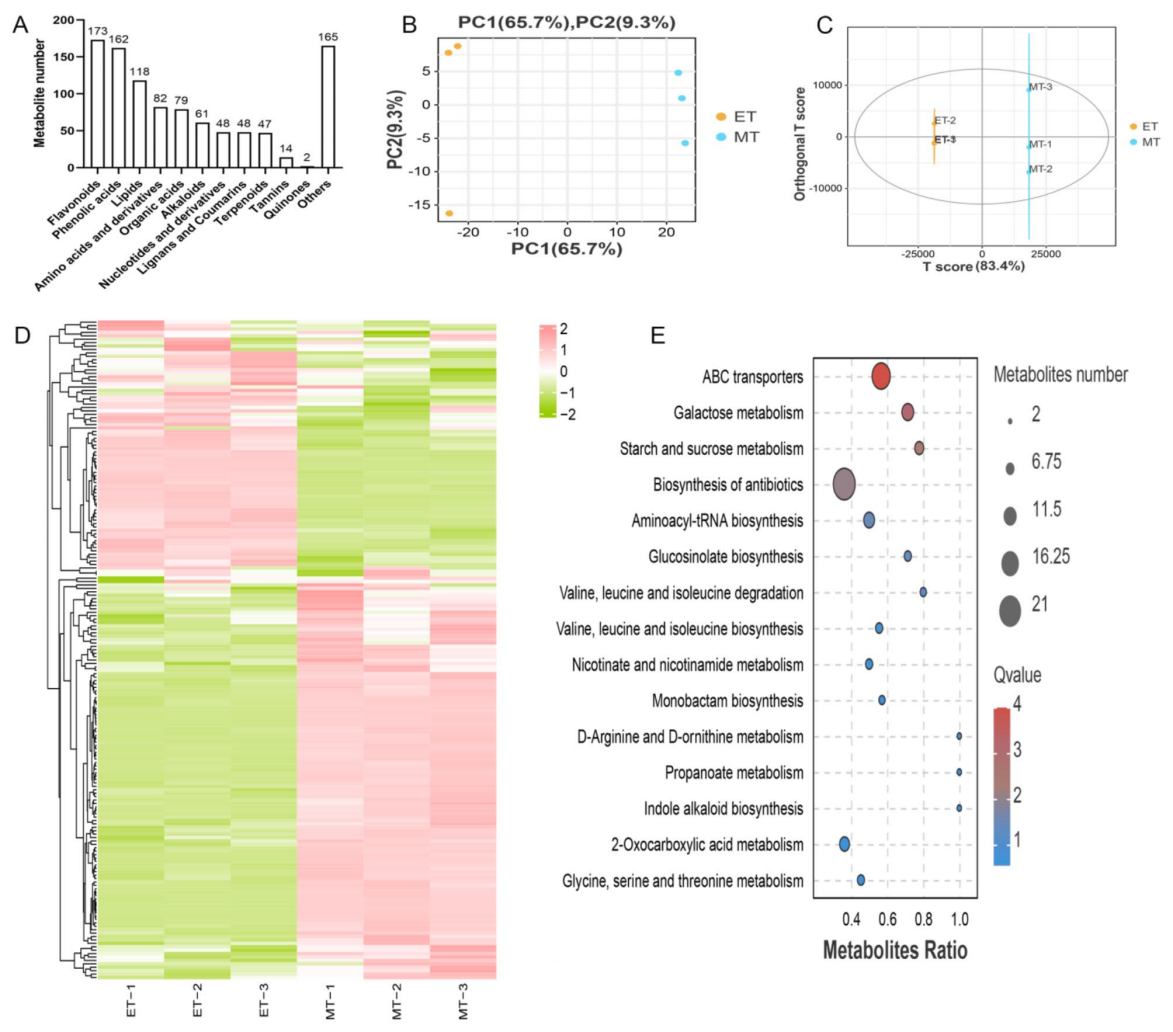
Metabolic analysis of sample relationship of metabolites. (A) Metabolites number. (B) Principal component analysis (PCA) at the initiation (ET) and expansion (MT) stages. (C) Orthogonal partial least-squares discriminant analysis (OPLS-DA) model at ET and MT stage. (D) A clustering heatmap of the differentially accumulated metabolites (DAMs). (E) KEGG enrichment pathway of DAMs.

To explore the metabolite differences of the initiation and expansion stages (ET and MT) in *C. kwangsiensis*, the tubers were analyzed using a non-targeted UHPLC-MS/MS with positive and negative modes. A heat map cluster showed good correlations among replicates which indicated high statistical repeatability of the data (Fig. S2A). A total of 999 metabolites were identified (Table S1), of which 12 category classifications were annotated according to their chemical properties (Fig. 1A) and these included flavonoids (173), phenolic acids (162), lipids (118), amino acids and their derivatives (82), organic acids (79), alkaloids (61), nucleotides and their derivatives (48), lignans and coumarins (48), terpenoids (47), tannins (14), quinones (2) and others (165). PCA and OPLS-DA model analysis were used to decrease the data dimensions and improve their interpretability and effectiveness. The PCA score plot showed that the two samples were divided into two sections (Fig. 1B), and the OPLS-DA results showed a high degree of distinction between the sample groups (Fig. 1C), suggesting that the metabolites could be effectively separated with differences between the initiation and expansion stages being evident.

According to the results of metabolomics analysis, 197 DAMs were found between the initiation and expansion stages, of which approximately two-thirds of DAMs were up-regulated (Fig. 1D). The metabolite differences significantly enriched metabolic pathways (Fig. 1E), including ABC transporters, galactose metabolism, starch and sucrose metabolism and biosynthesis of antibiotics. It is noteworthy that some important carbohydrate metabolites, secondary metabolites and amino acids in the initiation and expansion stages were significantly accumulated within pathway such as starch and sucrose metabolism, citrate cycle, galactose metabolism, phenylalanine metabolism, terpenoid compounds, ABC transporters, valine, leucine and isoleucine biosynthesis and degradation.

## Transcripts identification and analysis

**Fig. 2:**
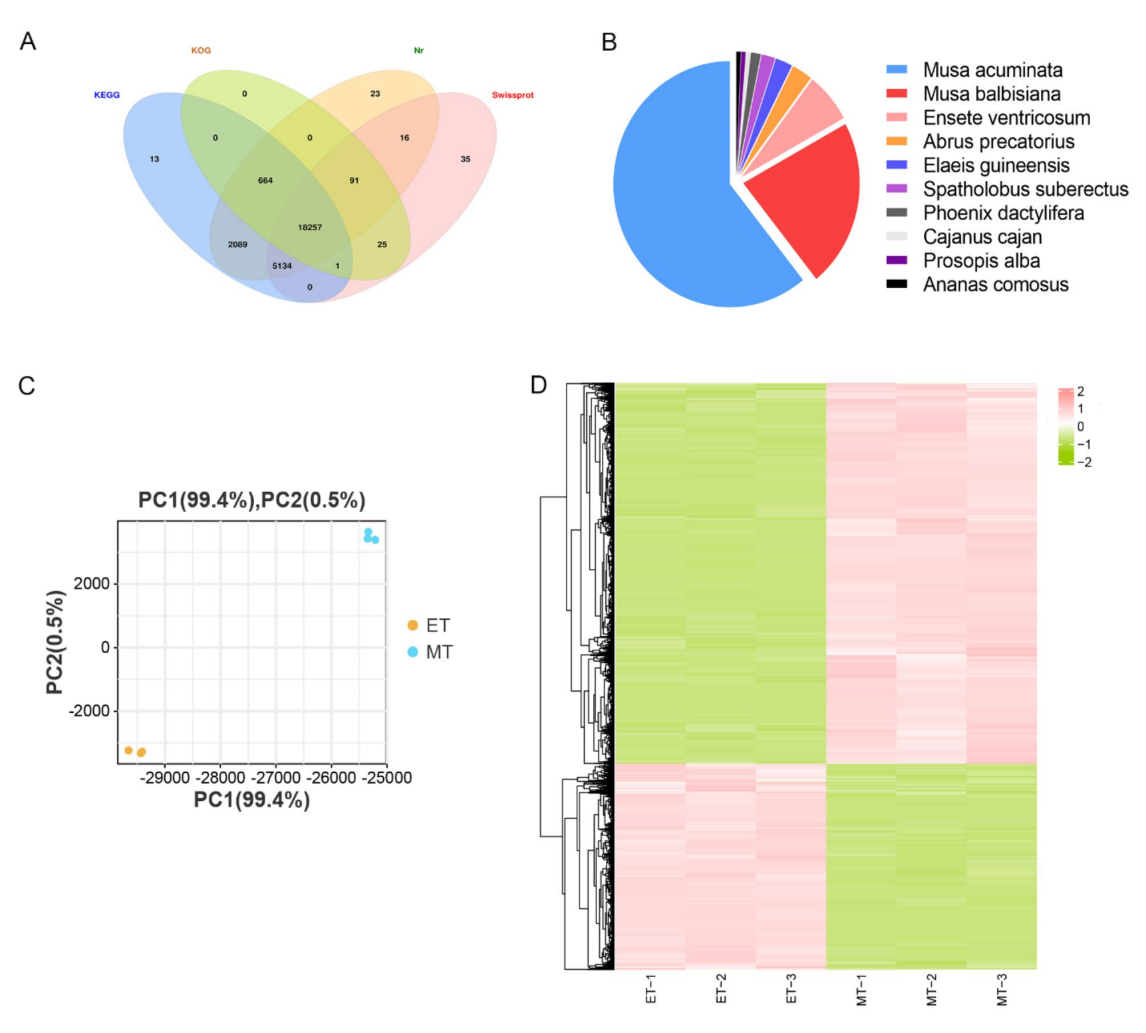
Transcriptomic analysis of the dynamic pattern of tuber expansion development. (A) Venn diagram annotated using four datasets. (B) Species distribution. (C) Principal component analysis of the sample relationship. (D) A clustering heatmap of the differentially expressed genes (DEGs).

The transcriptome library was constructed from a pool of mixed RNA consisting of the initiation and expansion stages (named EZ). A total of 31,889 transcripts and 26,590 genes were obtained (Table S2). The Pearson correlation coefficient analysis provided evidence of the biological consistency (Fig. S2B). 26,274, 26,158, 19,038 and 23,559 genes were functionally annotated with 4 functional databases NR, KEGG, KOG and SwissProt, respectively, making a total of 26,348 genes (Table S3). 18,257 genes were commonly annotated in NR, KOG, KEGG and SwissProt databases (Fig. 2A). Based on the functional annotation results of the NR database, the proportions of different species in the notes of genes were calculated, and 14,634 genes were aligned to *Musa acuminata* (Fig. 2B). To investigate the gene expression differences with *C. kwangsiensis* tubers, we conducted analyses at the initiation and expansion stages using six RNA-Seq libraries. PCA of gene expression levels demonstrated a distinct separation between samples from the initiation and expansion stages (Fig. 2C). After significance analysis, 6962 DEGs were identified, of which approximately two-thirds were up-regulated in the expansion stage (Fig. 2D).

**Fig. 3:**
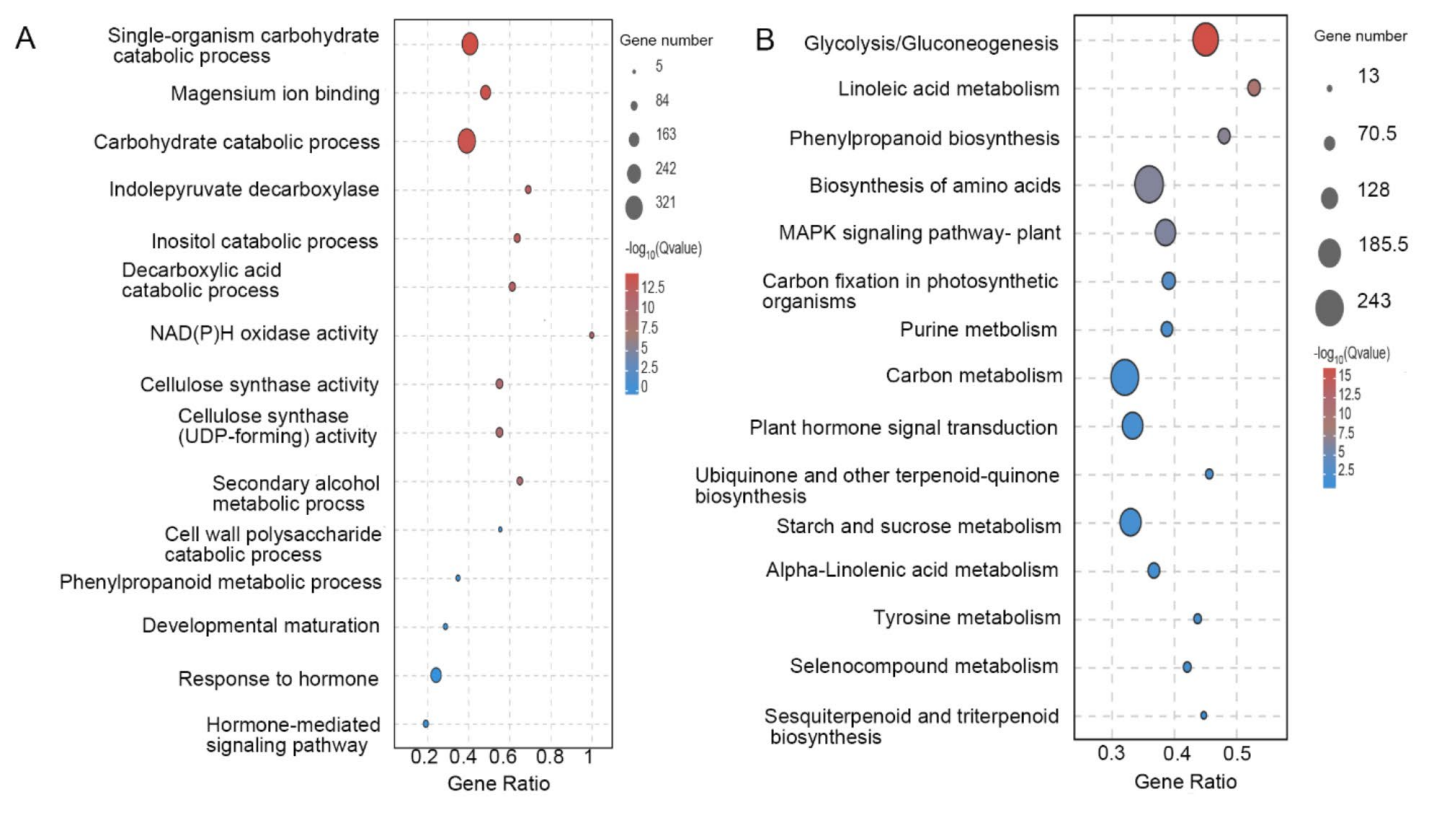
Identification analysis of the DEGs. (A) The top 15 pathways enriched in GO terms. (B) The top 15 pathways enriched in the KEGG pathway.

The enriched GO terms of DEGs in the paired groups ET-vs-MT, included carbohydrate catabolic process, NAD(P)H oxidase activity, cellulose synthase activity, cell wall polysaccharide catabolic process, and response to the hormone. In total, the unique gene families during tuber expansion were mainly related to energy functions, cell membrane composition, and cellular metabolic processes (Fig. 3A). The enriched pathways were hormone signal transduction, starch and sucrose metabolism, linoleic acid metabolism, MAPK signaling pathway and sesquiterpenoid and triterpenoid biosynthesis (Fig. 3B), suggesting several transcriptional regulations systems, as well as metabolite balance and environmental adaptation genes, were activated in *C. kwangsiensis* tuber expansion.

## Integrated analysis of the metabolites and genes

**Fig. 4:**
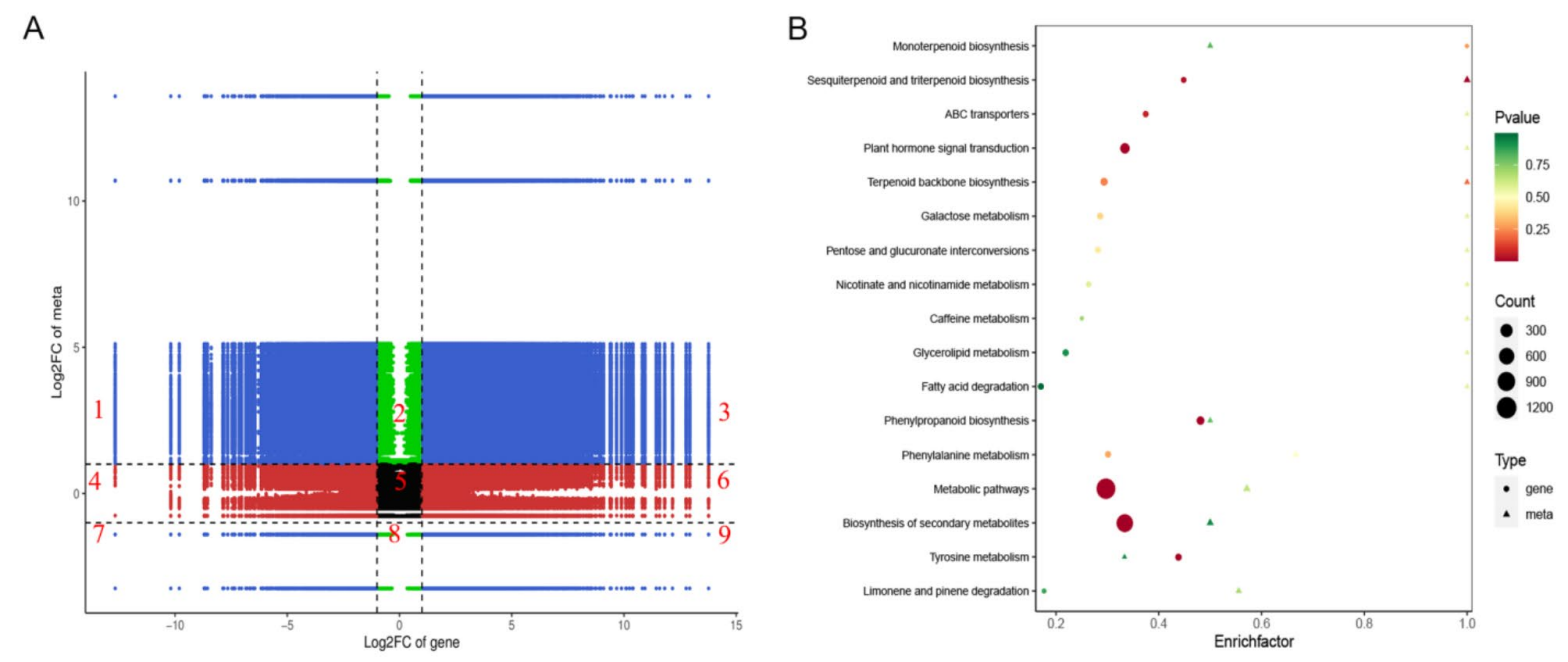
Integrated analysis of the DEGs and DAMs. (A) Correlation quadrant diagram analysis. Quadrant 5 shows unchanged DAMs and DEGs, quadrant 3 displays up-regulated DAMs and DEGs, and quadrant 7 exhibits down-regulated DAMs and DEGs. In quadrants 3 and 7, DAMs and DEGs are positively correlated with similar patterns. Quadrant 1 represents up-regulated DAMs and down-regulated DEGs, while quadrant 9 represents down-regulated DAMs and up-regulated DEGs. The DAMs and DEGs in quadrants 1 and 9 are negatively correlated with the opposite patterns. (B) KEGG enrichment analysis of DEGs and DAMs.

A Pearson correlation coefficients (PCC) > 0.8 was selected to analyze DAMs and DEGs by using Log2. The nine quadrant diagrams displayed a systematic view of variations in DAMs and DEGs in response to tuber expansion. As shown in quadrants 3 and 7, 1829 DEGs corresponding to 50 DAMs were positively correlated (Fig. 4A). They had similar consistent patterns, suggesting the change of metabolite accumulation may be regulated by genes in the tuber expansion stage. The enrichment pathways with DEGs and DAMs were displayed simultaneously in response to tuber expansion. It was found that DEGs and DAMs were simultaneously and significantly enriched in the pathways of terpenoid backbone, sesquiterpenoid, triterpenoid, monoterpenoid and phenylpropanoid biosynthesis as well as ABC transporters and plant hormone signal transduction (Fig. 4B).

## DEGs and DAMs related to plant hormone signal transduction

**Fig. 5:**
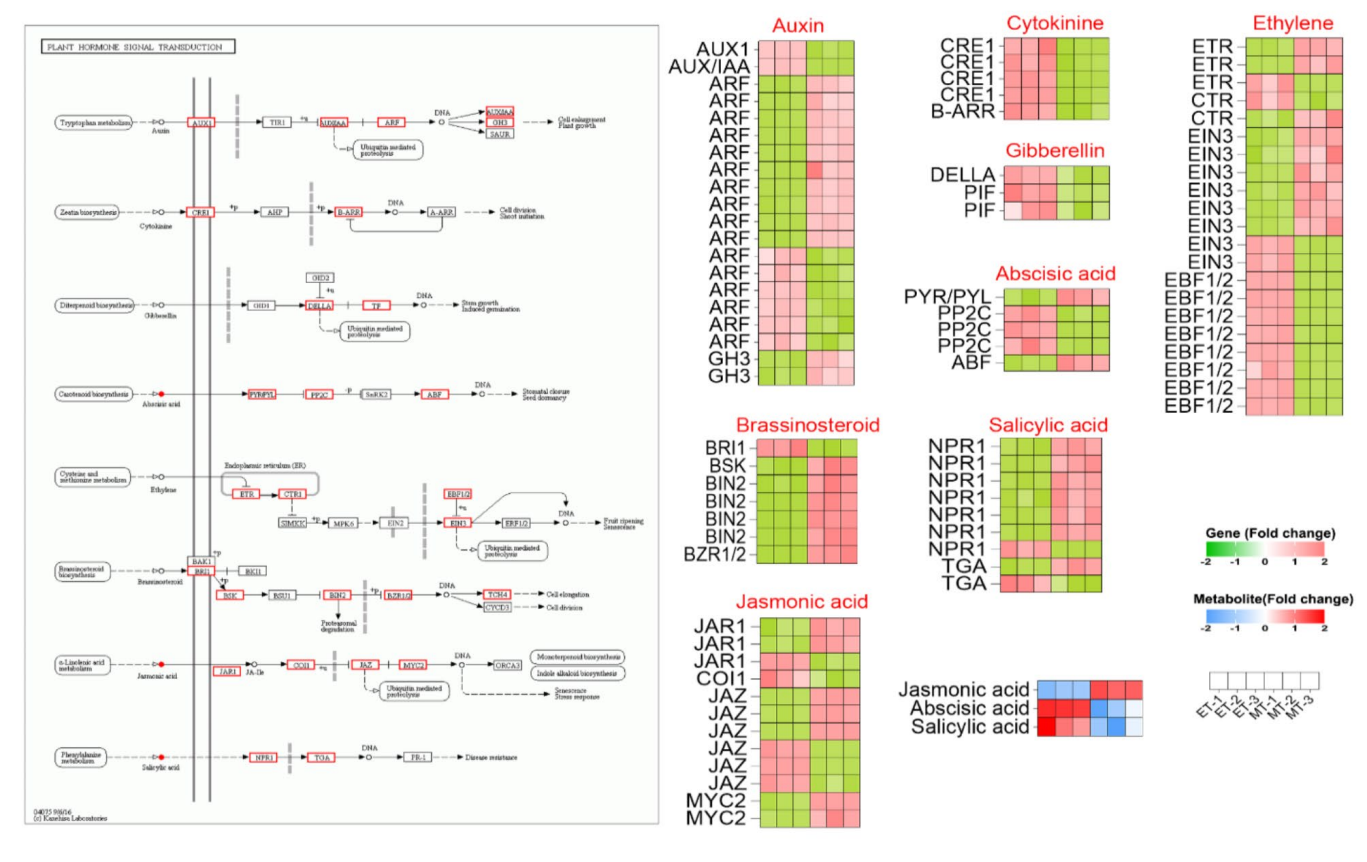
The DEGs and DAMs involved in plant hormone signal transduction pathway.

In this study, the plant hormone signal transduction pathway was enriched in the tuber expansion stage. Some DEGs were annotated to signal transduction pathways mediated by IAA, ZR, GA, ABA, ETH, BR, JA and SA, and with the majority exhibiting up-regulation in the tuber expansion stage (Fig. 5). Additionally, genes such as *ARF*, *EIN3* (ethylene-insensitive 3), *EBF1/2* (ein3-binding F-box 1 and 2), *BIN2* (brassinosteroid insensitive 2), *JAZ* (jasmonate ZIM-domain) and *NPR1* (non-expressor of pathogenesis-related genes 1), which are involved in the IAA, ETH, BR, JA and SA pathways were highly expressed. This suggests they potential critical role in facilitating the completion of tuber expansion. Meanwhile, hormone-related metabolites are also involved in these pathways, including JA, ABA and SA.

### DEGs and DAMs related to phenylpropanoid biosynthesis

**Fig. 6:**
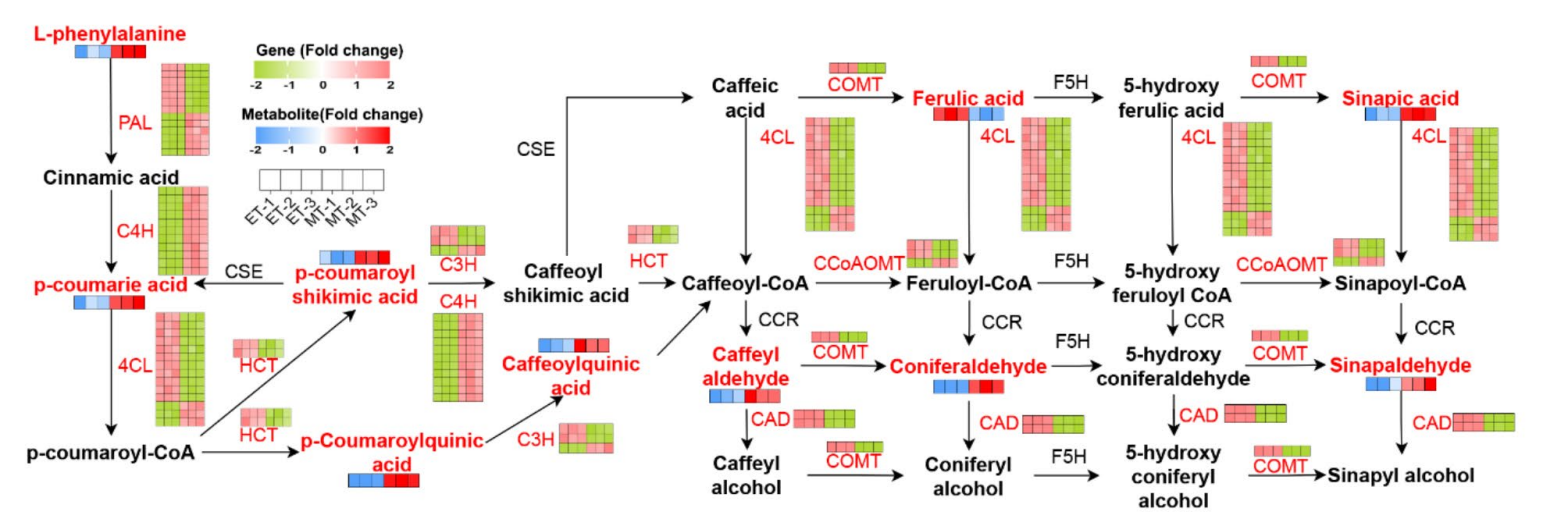
The DEGs and DAMs involved in phenylpropanoid biosynthesis.

In this study, the phenylpropanoid biosynthesis pathway was reconstructed with DEGs and DAMs of the tuber expansion stage (Fig. 6). Here, a total of 51 DEGs were assigned to this pathway. These DEGs included phenylalanine ammonia-lyase (*PAL*), 4-coumarate: CoA ligase (*4CL*), cinnamoyl CoA reductase (*CCR*), cinnamyl alcohol dehydrogenase (*CAD*), cinnamate 4-hydroxylase (*C4H*), coffee acyl coenzyme A-3-O-methyl transferase (*CCOAOMT*), shikimate O-hydroxycinnamoyl transferase (*HCT*), 2-C-methyl-D-erythritol-2,4-cyclodiphosphate synthase (*C3H*). In addition, most of DAMs involved in the phenylpropanoid biosynthesis pathway were up-accumulated at tuber expansion stage, including L-phenylalanine, p-coumaric acid, p-coumaroyl shikimate, p-coumaroylquinic acid, caffeoylquinic acid, caffeic aldehyde, coniferaldehyde, sinapinaldehyde and sinapic acid.

## DEGs and DAMs related to terpenoid biosynthesis

**Fig. 7:**
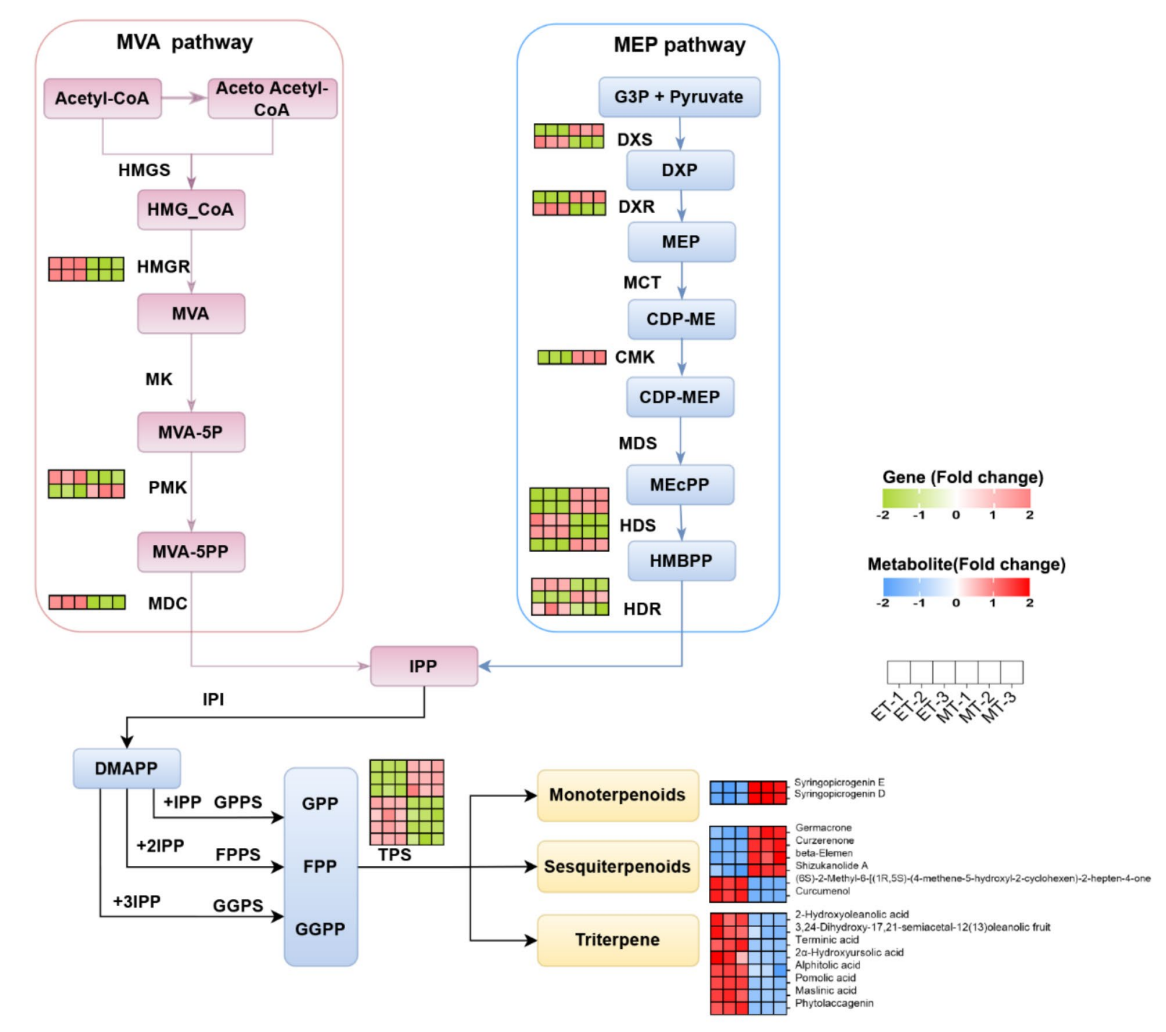
The DEGs and DAMs involved in terpenoid biosynthesis.

25 DEGs were found to be associated with the pathway of terpenoid backbone, sesquiterpenoid and triterpenoid biosynthesis in the tuber expansion stage (Fig. 7). Among them, one phospho-mevalonate kinase (*PMK*) gene, one *DXS* gene, one *DXR* gene, three (E)-4-hydroxy-3-methyl-but-2-enylpyrophosphate synthase (*HDS*) genes, one (E)-4-hydroxy-3-methylbut-2-enyl-pyrophosphate reductase (*HDR*) gene and three *TPS* genes were up-regulated, and two *HMGR* genes, one *PMK* gene, one 2-C-methyl-D-erythritol-2,4-cyclodiphosphate synthase (*MDC*) gene, one *DXS* gene, one *DXR* gene, two *HDS* genes, two *HDR* genes and four *TPS* genes were down-regulated. There were also 16 terpenoid metabolites categorized as monoterpenoids, sesquiterpenoids and triterpene. Among the monoterpenoids and triterpene metabolites, compounds such as syringopocrogenin E, syringopocrogenin D, 2-hydroxyoleanolic acid and phytolaccagenin exhibited reduced levels during the tuber expansion stage. In contrast, sesquiterpenoid metabolites such as germacrone, curzerenone and beta-elemen showed increased levels. These findings suggest that DEGs and DAMs associated with the terpenoid biosynthesis pathway play a role in tuber expansion.

## DEGs and DAMs related to ABC transporters

Among 18 *ABC* genes, *ABCB* and *ABCC* genes were related to 10 metabolic pathways according to the co-expressed network analysis (Fig. S3). *ABCB* and *ABCC* were linked to amino acids and nucleotides as well as their derivatives for the transport of metabolites. Also, certain secondary metabolites such as flavonoids, lignans and coumarins, tannins, alkaloid, terpenoids, organic acids and lipids showed connections in this network module.

### Transcription factors co-expression network analysis

**Fig. 8:**
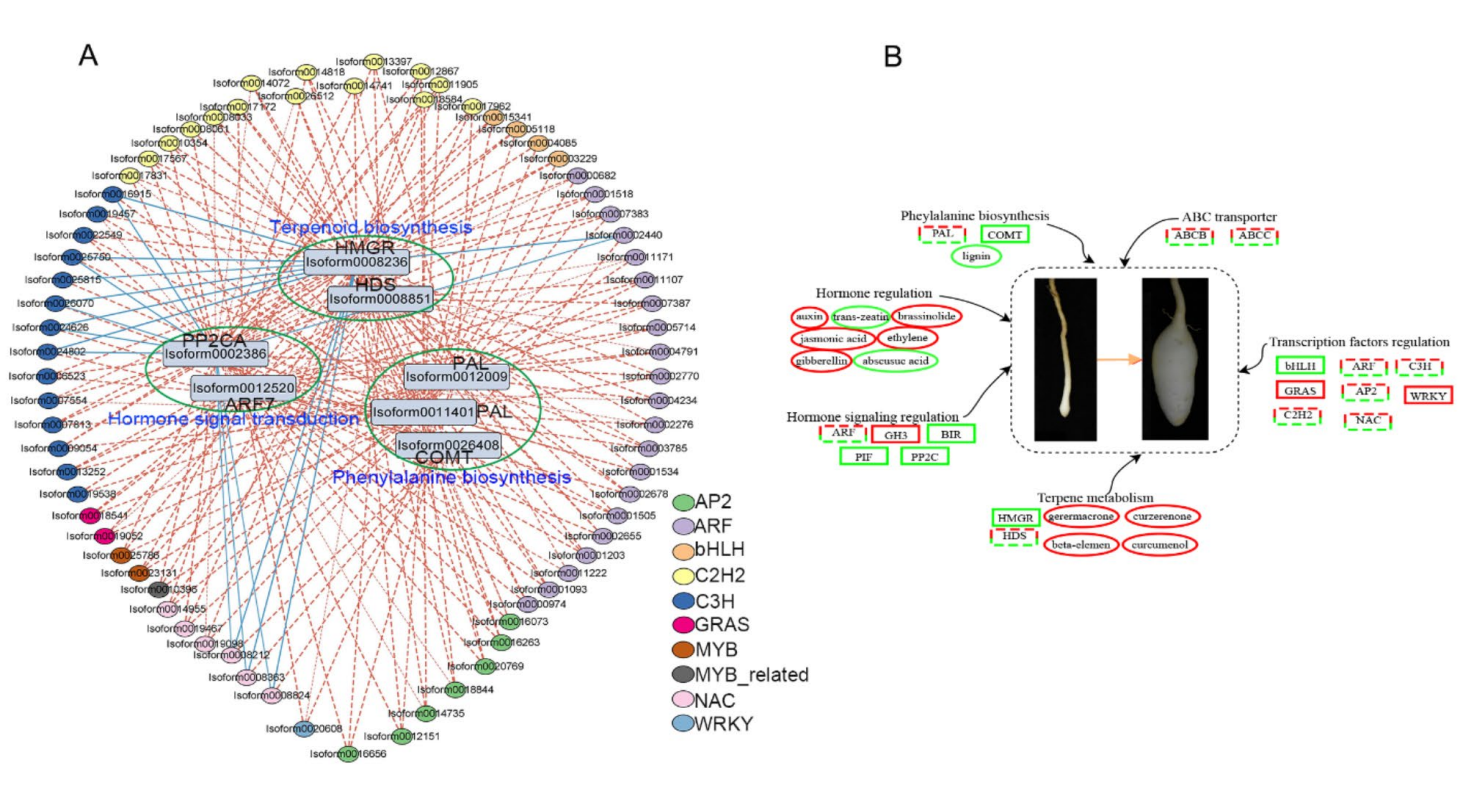
Network analysis of metabolomic and transcriptomic data. (A) Network analysis of TFs and screened DEGs. The dashed red lines represent positive correlations, and the solid blue lines represent negative correlations. (B) A putative model of the molecular network associated with tuber expansion. The rectangle represents the genes, and the ellipse represents the metabolites. The solid red lines represent up-regulated, the green solid lines represent down-regulated, and the dashed red-green lines represent up-regulated or down-regulated.

To further explore the regulatory mechanism underlying tuber expansion development in *C. kwangsiensis*, an analysis of TF interactions with key pathway genes association analysis was conducted to identify the core regulating TFs. Based on the metabolites and transcripts analysis data of DEGs in the three pathways, 24 differentially expressed TF families were screened and the co-expression network of TFs and key genes was constructed (Fig. 8A). In the molecular network diagram, the critical DEGs (*HDS*, *HMGR*, *ARF7*, *PP2CA*, *PAL* and *COMT*) were closely linked to the core TFs (*ARF*, *C2H2*, *C3H*, *NAC*, *bHLH*, *GRAS* and *WRKY*) and they were found to be embedded in the center of the network by hub genes screening.

## Discussion

The tubers of *C. kwangsiensis* are widely employed in traditional Chinese medicine to treat various ailments. The active ingredients of the tubers consist of a volatile oil composed of structurally diverse terpenoid compounds^2^, especially sesquiterpenoid, and the tuber expansion process is an important factor affecting the yield and quality formation of *C. kwangsiensis*^6^. Previous studies have focused on the sesquiterpenoid compound and pharmacological analysis of *C. kwangsiensis*^2,29,30^. A comparative transcriptome analysis of the rhizome tissues has previously been performed to investigate floral formation *C. kwangsiensis* under drought stress conditions^31^. Additionally, transcriptome profiling in *C.longa*, another species within the same genus, elucidated the involvement of phytohormone signaling and carbohydrate metabolism in the initiation of tuber formation^17^. However, little is known regarding the tuber development and metabolite accumulation in *C. kwangsiensis*. In this study, a systematically investigation into the molecular mechanisms underlying tuber expansion and metabolism accumulation was performed by selecting two different developmental stages of tuber formation (namely the initiation and expansion stages) for metabolomic and transcriptomic analysis. Over 999 metabolites and 26,590 genes were obtained from the samples and 197 DAMs and 6962 DEGs were identified. According to KEGG enrichment analysis, the metabolomic pathways related to carbohydrate metabolism, like starch and sucrose metabolism, citrate cycle, and galactose metabolism, were significantly enriched. These findings are similar to those observed in tuber formation of *C.longa*^17^, suggesting a potential relationship between tuber expansion and carbohydrate metabolism. Subsequently, an integrated analysis of metabolites and genes revealed a positive correlation between 50 DAMs and 1829 DEGs. According to the KEGG enrichment analysis of the DAMs and DEGs, pathways related to plant hormone signal transduction, phenylpropanoid biosynthesis, terpenoid backbone biosynthesis, sesquiterpenoid and triterpenoid biosynthesis and ABC transporters were involved. Furthermore, notable disparities were found in these DEGs and DAMs between the initiation and expansion stages. Based on our metabolomic and transcriptomic analysis, this study summarized a putative model of the molecular network associated with tuber expansion (Fig. 8B). Consequently, these pathways within the model were used to scrutinize the regulatory network governing tuber expansion.

### Hormonal signaling regulation

Hormones play a coordinating role in tuber and root growth and the development of crops and herbs. High levels of ABA can stimulate potato tuberization, while high levels of GA can regulate tuber elongation^9^. ZR, GAs, IAA and JA have been found to regulate the tuber enlargement of the yam^7^. High IAA, ZR and GA_3_ levels have been found at the rapid expansion stage of tuberous roots, suggesting that these compounds might promote tuber enlargement^32^. The concentration of ZR increased significantly during the adventitious root initiation stage^33^. The current study showed that a high level of ZR, ABA and SA appeared at the initiation stage of tuber development, while the levels of IAA, JA, GA_3_, ETH, and BR content increased during the tuber expansion stage (Fig. S1D). Thus, ZR, ABA and SA are beneficial for the tuber initiation, while IAA, JA, GA_3_, ETH and BR are beneficial for tuber expansion. Moreover, a total of 82 genes were detected in the plant hormone signal transduction pathways, including IAA, ZR, GA, ABA, ETH, BR, JA and SA pathways (Fig. 5). The majority of the *ARF* genes, along with two *GH3* genes, exhibited upregulation at the expansion stage, indicating they involvement in the regulation of the tuber expansion. This is consistent with the observed trend of the root swelling in *T. hemsleyanum*^27^. *DELLA* and *PIF3* gene levels were also identified as correlating with the hypocotyl length of Arabidopsis^34^. DELLAs were also regulated in the GA-mediated rhizome development process^35^, and high GA levels could reduce the transcription abundance of *DELLA* genes, suggesting that there was some feedback regulation^36^. Similar to these studies, GA_3_ level increased during tuber expansion development, while one *DELLA* gene and two *PIF* genes were down-regulated. In yam, the *JAR* (JA-amino acid synthetase), *JAZ* and *MYC2* family genes were highly expressed at the tuber rapid growth and expansion periods^26^, and most of the *JAR* and *JAZ* genes, along with two *MYC2* genes were upregulated at the expansion stage, indicating that these genes are involved in regulating the tuber expansion development. High *ABF* expression levels were reported to inhibit potato tuber formation and yam tuber development^15,26^. The expression level of the *ABF* gene was upregulated in the expansion stage, which also indicated that the *ABF* gene played a negative role in tuber expansion. The type-B ARABIDOPSIS RESPONSE REGULATORS (*B-ARR*) gene can target with WUSCHEL (a key gene required for apical meristem maintenance) gene to impact shoot development^37^. One B-ARR gene was detected in the ZR pathway, which was highly expressed in the initiation stage, suggesting this gene can regulate the initiation stage of tuber development. In summary, ZR, ABA and SA are beneficial for tuber initiation, while IAA, JA, GA_3_, ETH and BR are beneficial for tuber expansion. *ARF*, *GH3*, *DELLA*, *PIF*, *JAR*, *JAZ*, *MYC2*, *ABF* and *B-ARR* genes may regulate the changes of hormone signaling pathways during tuber expansion of *C. kwangsiensis*.

### Phenylalanine biosynthesis regulation

Lignin, as the primary constituent of the cell wall, plays a vital role in providing essential structural support during plant growth, development, and defense processes. Lignin is produced via the phenylalanine biosynthesis pathway in plant cells, and *PAL*, *C4H*, *4CL*, *HCT*, *COMT* and *CAD* genes have been reported to be key enzymes for lignin biosynthesis^38–41^. In this study, a total of 51 genes were detected in the phenylpropanoid biosynthesis pathway, including the ones listed above (Fig. 6). Previous studies have shown that the expression of the *CAD* gene decreased in Arabidopsis resulting in a reduction of lignin content^40^, and silencing of the *COMT* gene in alfalfa resulted in reduced content of G-lignin^42^. Similarly, the expression levels of *COMT* and *CAD* genes were decreased during tuber expansion development in *C*. *kwangsiensis*, resulting in a decrease in lignin during this process. Similar to these studies, the expression of lignin-related genes was reduced at the root swelling stage in sweet potato and *T. hemsleyanum*^27,43^. We speculate that lignin is needed to construct fibrous roots in the initiation stage and that the lignin decreases as the carbohydrate and secondary metabolites increase during the expansion stage.

### Terpenoid metabolites regulation

In the current study, monoterpenoid, sesquiterpenoid, and triterpenoid metabolites and 25 genes were detected in the terpenoid biosynthesis pathway during the tuber expansion development. The biosynthesis of terpenoids occurs via the MVA and MEP pathways. In this study, two *HMGR* genes, one *DXS* gene and one *DXR* gene were downregulated during the tuber expansion development, while other *DXS* and *DXR* genes were upregulated (Fig. 7). These genes have been reported to be key enzymes and function in the biosynthetic of terpenoids^20^. In addition, TPSs can catalyze the conversion of GPP, FPP and GGPP into monoterpenes, sesquiterpenes and diterpenes, respectively, which can be categorized as monoterpene, sesquiterpene and diterpene synthases^44^. Moreover, *PhTPS1* can increase the content of sesquiterpenes and regulate seed formation^45^. In this study, the expression of *TPS* genes was also correlated with terpenoid content, indicating that they play a key role in terpenoid biosynthesis. Many studies have proposed that four sesquiterpenoids (germacrone, curzerenone, beta-elemen and curcumenol) were active components in the volatile oils of *C. kwangsiensis* tuber^2,3^. Sesquiterpene volatiles can affect reproductive organ development and seed yield in *Petunia hybrida*^45^. Germacrone, curzerenone, beta-elemen and curcumenol metabolites were abundantly accumulated during the tuber expansion development, suggesting these sesquiterpene metabolites can affect the tuber expansion of *C. kwangsiensis*.

### ABC transporters regulation

The ABC transporters participate in various biological processes and play essential roles in the transmembrane transport of secondary metabolites^46^. In this study, 18 *ABC* genes were shown to be involved in 10 metabolic pathways (Fig. S3), and these genes were abundant in expression during tuber expansion development. ABC transporter genes have been reported to be involved in terpenoid metabolites. Some ABC families in *Salvia miltiorrhiza*, such as *ABCB1*, *ABCB9*, *ABCC1*, *ABCC10*, *ABCD3* and *ABCG2* genes were identified as regulating the transport and accumulation of tanshinone^47^. Seven ABC transporter-encoding transcripts were abundantly expressed in different tissues, and involved in long distance and transport of picrosides in *Picrorhiza kurroa* organs^48^. In this study, four *ABCB* (Isoform0000383, Isoform0000823, Isoform0001225 and Isoform0000924) and seven *ABCC* (Isoform0001855, Isoform0002772, Isoform0000254, Isoform0000318, Isoform0005480, Isoform0007637 and Isoform0009011) genes were highly co-expressed with four terpenoids (maslinic acid, mws1610, alphitolic acid, Lmzn106284, curcumenol, MWSmce591 and germacrone, MWSmce564). Furthermore, the *AtABCG14* was involved in cytokinin biosynthesis and transported cytokinin from the roots to the shoots^49^. In the present study, one *ABCB* gene (Isoform0001239) and two *ABCC* genes (Isoform0000125, Isoform000062) were highly co-expressed with JA (pme1654). These results show that ABCB and ABCC genes are involved in terpenoids (maslinic acid, maslinic acid, curcumenol, germacrone) and JA hormone transport during the tuber expansion development of *C. kwangsiensis*.

### Transcription factors regulation

In the current study, the hormone signal transduction, phenylpropanoid and terpenoid biosynthesis pathways of DEGs and DAMs were used to accurately determine the critical point during the transition from expansion in tubers, such that, performing multi-omics integrative analysis, these pathway hub genes identified the TFs from the constructed co-expression network. These hub genes, including *HMGR*, *HDS*, *PP2CA*, *ARF7*, *PAL* and *COMT* genes, were found to be associated with several TF families (*ARF*, *C3H*, *C2H2*, *NAC*, *AP2*, *bHLH*, *GRAS*, *WRKY*, *MYB* and *MYB*_related) in the co-expression network (Fig. 8A). *HMGR5*, *HDS1* and *FPPS3* genes were regulated by b*HLH20*, *bHLH9*, *ERF21*, *NAC4*, *bHLH5*, *WRKY7* and *WRKY8*, which were previously identified in *Agriophyllum squarrosum*^23^. The *NAC*, *HB*, *MYB*, and *WRKY*, are lignin synthesis-associated TFs, and these possibly combine with *C3H2*, *CCoAOMT*, *COMT* and other genes involved in phenylpropanoid and lignin biosynthetic processes^50,51^. The 14 TF-encoding DEGs, such as *WRKY*, *bHLH*, *GRAS*, *NAC* and *ARF*, responded to hormone signal regulation and were found to regulate the accumulation of terpenoids in celery^24^. *ARF*, *C3H*, *C2H2*, *NAC*, *AP2*, *bHLH*, *GRAS*, *WRKY*, *MYB* and *MYB*_related family genes were suggested to respond to the hub genes of hormone signal transduction, phenylpropanoid and terpenoid biosynthesis pathways based on the co-expression network. Therefore, it can be inferred that these DEGs are involved in tuber expansion development in *C. kwangsiensis*.

## Conclusion

The mechanism of tuber expansion development was studied by metabolomic and transcriptomic analysis of *C. kwangsiensis*. The hormone levels played coordinating roles in the tuber expansion development. Related candidate genes were detected, including *ARF*, *GH3*, *DELLA*, *PIF*, *BRI1* and *PP2CA*. The phenylalanine biosynthesis pathway is an important way to produce lignin, and *PAL* and *CCOM* genes were significantly expressed. The *HMGR* and *HDS* genes, and sesquiterpene metabolites (gerermacrone, curzerenone, beta-elemen and curcumenol) accumulated during the tuber expansion development. Some ABC transporter genes and TFs were predominantly involved in tuber expansion development. These findings will provide a foundation-based mechanism for tuber expansion in *C. kwangsiensis* and will aid the cultivation of this type of medicinal plant.

### Methods

#### Plant material

The tubers of *C. kwangsiensis* plants were planted in an experimental field (Guangxi Botanical Garden of Medicinal Plants) in Nanning City, Guangxi Province China (Fig. S1A, B). The experimental materials were gathered at 60 (tuber initiation stage) and 210 days (tuber expansion stage) after planting. The experimental material of the ten plants was mixed as a biological replicate and immediately quickly frozen in liquid nitrogen.

### Determination of endogenous hormone

The hormone of tubers was extracted with 75% methanol containing 5% formic acid. The homogenates were then centrifuged for 10 min at 6000 rpm at 4℃ and the supernatants were collected. After concentrating at 35℃, the resulting dry residue was re-dissolved in 0.1 mL of 80% methanol. The levels of indole-3-acetic acid (IAA), jasmonate (JA), gibberellin (GA_3_), ethylene (ETH), salicylic acid (SA), brassinosteroid (BR), zeatin riboside (ZR) and abscisic acid (ABA) in tubers were determined with an ultra-performance liquid chromatography-tandem mass apectrometry (UHPLC-MS) system as previously described^7^.

### Metabolomic profiling

Metabolomic analysis was conducted on tuber samples collected at two distinct developmental stages, and with each stage the determinations were replicated three times for robustness. Samples underwent initial preparation by immersion in 1.0 mL of 70% aqueous methanol at 4℃ overnight. Subsequently, extracts were processed through solid-phase extraction cartridges and filtered using a 0.22 μm pore size microporous membrane before LC-MS analysis. Utilizing a UPLC-MS system, chromatographic separation was achieved by employing a Waters C18 column with mobile phases comprising 0.04% acetic acid in water (Phase A) and 0.05% acetic acid in acetonitrile (Phase B) at 40 °C. The solvent gradient transitioned linearly over 15 min, ranging from 95:5 Phase A/Phase B to 5:95 Phase A/Phase B and returning to the initial conditions. The flow rate was maintained at 0.4 mL/min, with a 2 µL injection volume. High-resolution mass spectra were acquired in the positive ion mode using electrospray ionization. Data processing encompassed filtering, peak detection, alignment and calculations facilitated by Analyst 1.6.1 software. Metabolite identification involved accessing internal and public databases such as MassBank, KNApSAcK, HMDB, MoTo DB, and METLIN. Principal component analysis (PCA) and orthogonal partial least squares discriminant analysis (OPLS-DA) were employed to discern the metabolites with significantly distinct levels (p-value < 0.05). The differentially altered metabolites (DAMs) were identified based on a log2 fold change (FC) ≥ 2 or p-value ≤ 0.5 and variable importance in projection (VIP) scores > 1. Finally, metabolites were subjected to pathway analysis utilizing KEGG^52^ and MetaboAnalyst 4.0 software for comprehensive elucidation.

#### RNA extraction, isoform sequencing and Illuminea sequencing

The total RNA from two different developmental stages of tuber formation was extracted using the Trizol™ reagent kit (Invitrogen, Carlsbad, CA, USA) by following the manufacturer’s instructions. The purity, concentration and integrity of the RNA samples were assessed by using a NanoDrop micro-spectrophotometer (Thermo Scientific) and Agilent 2100 Bioanalyzer. To obtain an accurate reference for the genes in the *C. kwangsiensis* plant, full-length transcriptome sequencing was performed. Total RNA from two tissue samples was uniformly combined to create an Iso-Seq library by following the Clontech SMARTer PCR cDNA Synthesis Kit protocol and then sequenced on the PacBio SequelII platform (Gene Denovo Biotechnology Co. Ltd., Guangzhou, China). Primary Iso-Seq data were processed using SMRTlink v5.0.1 software to generate read sequences, and circular consensus sequences were derived following error correction. Sequences were then classified into non-full-length and full-length sequences based on 5’primers, 3’primers and polyA structures. The clustering of full-length sequences facilitated the extraction of cluster consensus sequences, which were refined to obtain full-length consensus sequences for subsequent analysis.

The sequence annotation of gene function was performed with NR (NCBI non-redundant protein database, https://www.ncbi.nlm.nih.gov/), Pfam (http://pfam.xfam.org/), NT (NCBI nonredundant nucleotide sequences), KO (Kyoto Encyclopedia for Genes and Genomes (KEGG) database, https://www.genome.jp/kegg)^52^, GO (Gene Ontology), Swiss-Prot (http://www.expasy.ch/sprot), and KOG/COG (http://www.ncbi.nlm.nih.gov/COG/) to obtain a refined gene reference sequence (subsequently named EZ).

### High throughput transcriptomic analysis

Individual tuber stage total RNA samples were utilized to construct Illumina sequencing cDNA libraries by using the NEBNext^®^ UltraTM RNA Library Prep Kit for Illumina, with subsequent RNA-seq conducted on the Novaseq 6000 platform in paired-end mode platform (Illumina, Gene Denovo Biotechnology Co. Ltd., Guangzhou, China). A total of six sets of transcriptome raw data were obtained. FASTP V0.18.0 was applied to the data for quality control. Bowtie2 V2.2.8 was used for read assembly, and the resulting clean reads were used to calculate gene abundance. The clean reads were mapped to the reference transcriptome sequence (named EZ) by TOPHAT V2.0.9. Gene expression levels were quantified using fragments per kilobase of transcript per million mapped reads (FPKM). The differentially expressed genes (DEGs) were identified with the NOISeq method, with criteria set at |log2 (fold change) | > 2 and a statistically significant p-value < 0.05. GO enrichment and KEGG pathway enrichment analysis were conducted, with significance determined at a corrected p-value threshold ≤ 0.05, considering the enrichment factor.

#### Correlation network analysis of the transcriptomic and metabolomic data

To elucidate the data relationships, normalization and statistical analyses were applied to both transcriptome and metabolome datasets. Functional analysis, metabolic pathway enrichment, and correlation analysis were subsequently employed to identify pivotal genes, metabolites, and pathways. Pearson correlation analysis between the DEGs and DAMs utilized the normalized data in the R language. Correlation and KEGG enrichment analyses were based on Pearson Correlation Coefficient (PCC) values with |PCC| ≥ 0.8 for both DEGs and DAMs. Additionally, a network diagram depicting the interplay between genes and metabolites was constructed using CYTOSCAPE V.3.7.2.

#### Quantitative real-time PCR (qRT-PCR) analysis

To validate the results of RNA-seq, 14 DEGs were selected for the qRT-PCR analysis, while the actin gene was used as an internal reference. The sequence primers were designed by Primer 5.0 (Table S4). The total RNA of the samples was extracted and reverse-transcribed into cDNA with three technical replicates for each biological replicate. qRT-PCR was performed using the LightCycler 96 instrument (Roche, Basel, Switzerland), with reaction conditions of 95 ℃ for 5 s, 60 ℃ for 10 s, and 72 ℃ for 15 s for 40 cycles. According to a previous study^53^, the 2^–ΔΔCt^ method was employed to calculate the relative expression levels of the mRNAs. ACTIN was used as the reference gene (Table S4), the initiation stage was used as the control sample. When the transcriptomic and qRT-PCR data were combined, the candidate genes showed similar patterns of expression (Table S4).

## Electronic supplementary material

Below is the link to the electronic supplementary material.

Supplementary Material 1

Supplementary Material 2

## Data Availability

The datasets for this study can be found in the National Center of Biotechnology Information with the BioProject accession code PRJNA1089530 (https://www.ncbi.nlm.nih.gov/bioproject/PRJNA1089530/). This published article and its supplementary information files include all data generated or analyzed in this work.
