# Supplementary material for: Metabolic and transcriptional analysis of tuber expansion in Curcuma kwangsiensis
Source: Sci Rep. 2025 Jan 10;15:1588. doi: 10.1038/s41598-024-84763-9 (PMC11724066; doi:10.1038/s41598-024-84763-9)
Supplement: Supplementary file 2 — Supplementary Material 2 [file 41598_2024_84763_MOESM2_ESM.docx]

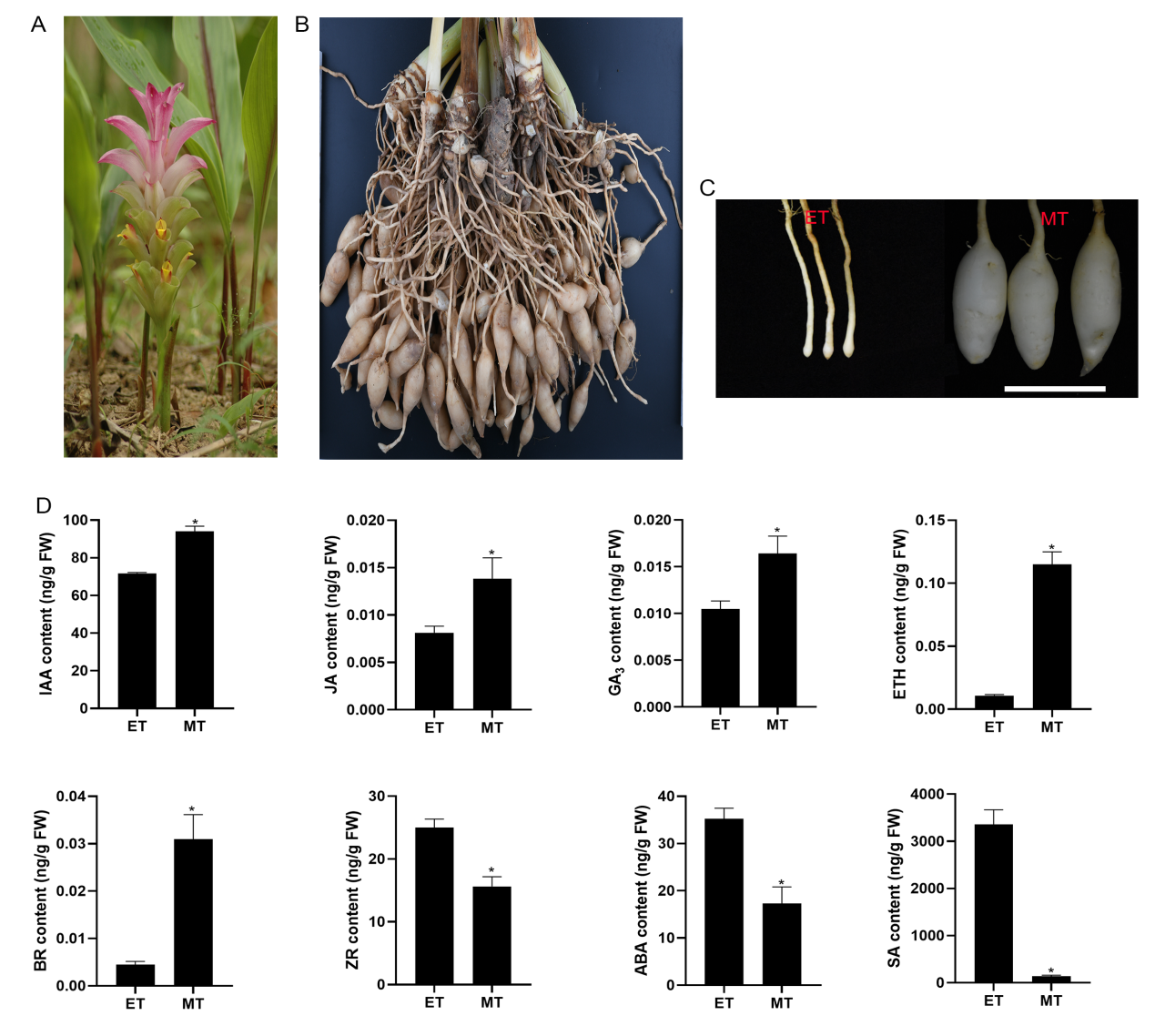


Figure S1 Phenotypic (A-C) and hormone levels (D) during tuber expansion development of *C. kwangsiensis*. The asterisks in the bar plots indicate significant differences from the initiation stage (ET) based on Students t-test (p < 0.05).


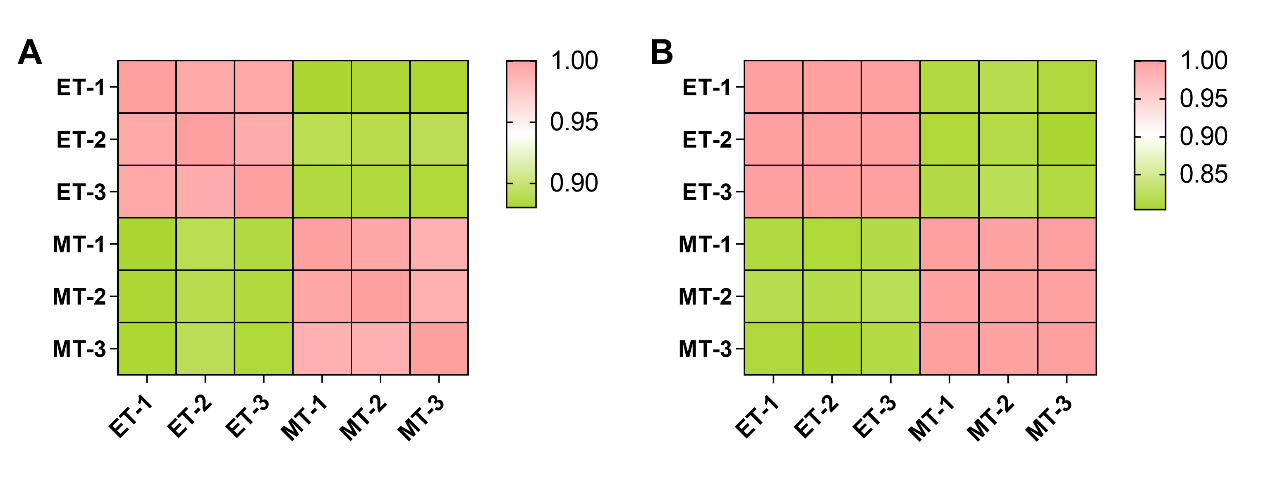


Figure S2 The pearson correlation coefficient analysis of metabolomic (A) and transcriptomic (B) data.


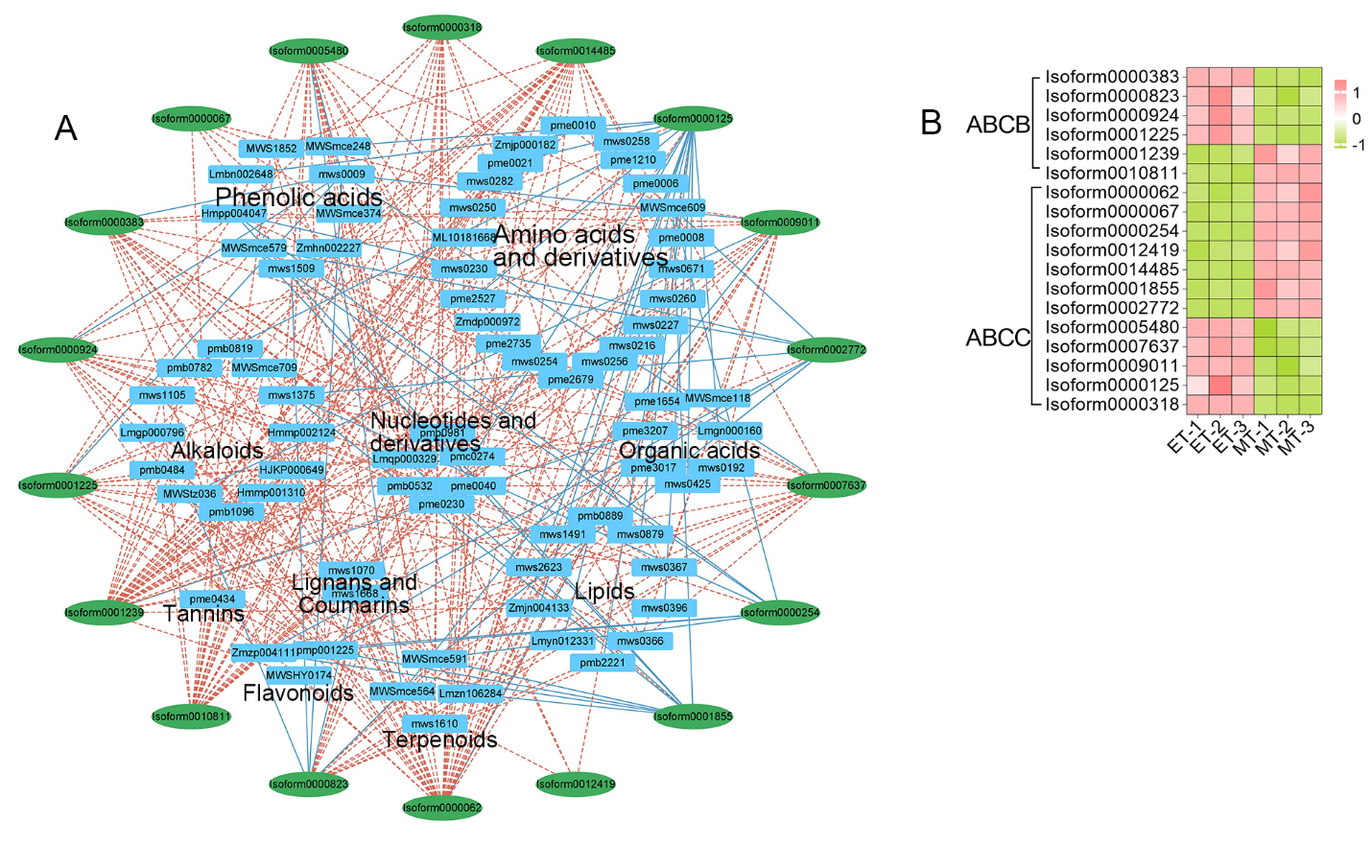


Figure S3 The DEGs and DAMs involved in ABC transporters. (A) The network analysis of ABC genes and metabolites. The dashed red lines represent positive correlations, and the solid blue lines represent negative correlations. (B) The expression level of ABC genes.
